# Anti-inflammatory effect of a pimarane diterpenoid isolated from *Nepeta adenophyta* Hedge based on a network analysis approach and experimental assessment

**DOI:** 10.3389/fphar.2025.1652902

**Published:** 2025-12-18

**Authors:** Wei Zhao, Meng Li, Chuanqing Jia, Iftikhar Ali, Long Chen

**Affiliations:** 1 The Second Affiliated Hospital of Heilongjiang University of Chinese Medicine, Harbin, China; 2 Shandong Academy of Chinese Medicine, Jinan, China; 3 Department of Chemistry, Karakoram International University, Gilgit, Pakistan; 4 Key Laboratory for Natural Active Pharmaceutical Constituents Research in Universities of Shandong Province, School of Pharmaceutical Sciences, Qilu University of Technology (Shandong Academy of Sciences), Jinan, China

**Keywords:** diterpene, Nepeta, network analysis, lipopolysaccharide-induced inflammation, RAW264.7 cell line, NF-κB and PPARγ signaling pathways

## Abstract

**Background:**

As an important folk medicine in Pakistan, *Nepeta adenophyta* Hedge has been widely used to treat abdominal pain, kidney pain, headaches, and for the alleviation of dysmenorrhea. The pimarane-type diterpenoids are known for their anti-inflammatory activity, but their mechanistic pathways remain understudied. 2,4b,8,8-Tetramethyl-2-vinyl-1,2,3,4,4a,4b,5,6,7,8,8a,9-dodecahydro-phenanthrene-3,5-diol (**1**), a pimarane diterpenoid, was detected primarily from *N. adenophyta* Hedge through its gas chromatography-mass spectrometry (GC-MS) fragmentation pathways. The GC-MS-guided isolation yielded compound **1** (NAC, *N. adenophyta* compound) in pure form.

**Methods:**

The GC-MS guided isolation of compound **1** was performed by column chromatography on normal silica gel. The structure was characterized by spectroscopic techniques. Then, the potential targets, pathways, and hub genes for treating inflammatory diseases were screened out through network analysis, and core targets were docked with **1** via docking software. Based on the results of network analysis, an MTT assay was performed to determine cell proliferation in the RAW264.7 cell line. Inflammatory cytokines, such as tumor necrosis factor-alpha (TNF-α), interleukin-1 beta (IL-1β), interleukin-6 (IL-6), and prostaglandin E2 (PGE2), were tested by enzyme-linked immunosorbent assay (ELISA). Immunofluorescence and Western blot assays were used to verify the function of **1** in the treatment of inflammation.

**Results:**

Compound **1** was isolated from *N. adenophyta* Hedge in its pure form. The pharmaceutical network results showed that it has a potential anti-inflammatory effect through the PPAR and NF-κB signaling pathways. The ELISA results showed that **1** could attenuate the content of pro-inflammatory cytokines. Additionally, the translocation of NF-κB p65 into the nucleus was significantly decreased in the immunofluorescence method. The Western blot analysis results showed that **1** significantly inhibited the protein expression of inducible nitric oxide synthase (iNOS) and cyclooxygenase-2 (COX-2). Furthermore, it decreased the phosphorylation of nuclear factor kappa B inhibitor α (IκBα) and toll-like receptor 4 (TLR4) by the NF-κB signaling pathway. Compound **1** also reduced reactive oxygen species (ROS) levels and restored overexpressed heme oxygenase-1 (HO-1) and serine/threonine kinase (AKT) to the basal level.

**Conclusion:**

The present study indicates that compound **1** shows a significant anti-inflammatory effect, potentially through intervention in the NF-κB and PPARγ signaling pathways.

## 1 Introduction

Inflammation is a complex physiological and pathological process caused by harmful stimuli in the internal and external environment. It eliminates harmful substances and damaged cells by utilizing specific signaling pathways that significantly contribute to the progression of many human diseases (Hwang et al., 2019). Lipopolysaccharide (LPS), a component in the cell wall of Gram-negative bacteria, is a strong inducer of inflammation and immune responses in the body (Hu et al., 2021). It is the most powerful activator of mononucleated macrophages and plays a crucial role in triggering the inflammatory and immune response of the host against bacterial infection. After stimulation, macrophages can secrete a variety of inflammatory mediators, such as nitric oxide (NO), prostaglandin E2 (PGE2), inducible nitric oxide synthase (iNOS), cyclooxygenase-2 (COX-2), tumor necrosis factor-α (TNF-α), interleukin-6 (IL-6), and interleukin-1β (IL-1β) (Kim et al., 2019). High production of these inflammatory mediators can lead to systemic inflammatory response syndrome, severe tissue damage, and endotoxic shock (Gao et al., 2020). Therefore, activated macrophage cells have been widely used to evaluate the anti-inflammatory effects of various drugs.

Nuclear factor kappa B (NF-κB) constitutes a family of nuclear transcription factors with a variety of biological activities. Some exogenous stimulant substances easily induce and activate NF-κB. By specifically binding to the promoter gene sites, NF-κB initiates the transcription of downstream genes and the expression of promoting proteins, thus accelerating the synthesis and secretion of inflammation-related factors, leading to a series of changes in the body and mediating the onset of related diseases (Chen et al., 2011). NF-κB is shown to be one of the upstream signals of the inflammatory cascade. By inhibiting the NF-κB signal, the levels of iNOS and COX-2 in RAW264.7 cells could be downregulated, and the secretion of inflammatory mediators could be reduced (Wen et al., 2020; Linghu et al., 2020). This inhibition is mainly mediated by intervention in the NF-κB signaling cascade and subsequent p65 translocation from the cytoplasm to the nucleus (Kim et al., 2020). Some researchers have proved that the upstream kinases, such as extracellular signal-regulated kinase (ERK) and PI3K/AKT, are also implicated in increasing transcriptional activity of NF-κB. Thus, NF-κB plays a core role in mediating inflammation and immune responses (Guha and Mackman, 2001).

The anti-inflammatory potential of certain plant extracts and their pure natural products has been reported (Olanlokun et al., 2021; Yimer et al., 2020; Zheng et al., 2021; Tekulu et al., 2020). *Nepeta* species are reported to exhibit good anti-inflammatory activity. *Nepeta adenophyta* Hedge (Lamiaceae) has been reported to treat abdominal pain, menstrual pain, and control bleeding disorders in Astore, Gilgit (Pakistan). *Nepeta adenophyta* Hedge (*Nepeta adenophyta*) has also shown potent analgesic and anti-inflammatory effects (Ali et al., 2021). In the present study, a pimarane diterpenoid, namely, 2,4b,8,8-tetramethyl-2-vinyl-1,2,3,4,4a,4b,5,6,7,8,8a,9-dodecahydro-phenanthrene-3,5-diol (**1**, NAC, *N. adenophyta* compound), was isolated efficiently from *N. adenophyta* Hedge via gas chromatography-mass spectrometry (GC-MS) guided isolation.

**FIGURE 1:**
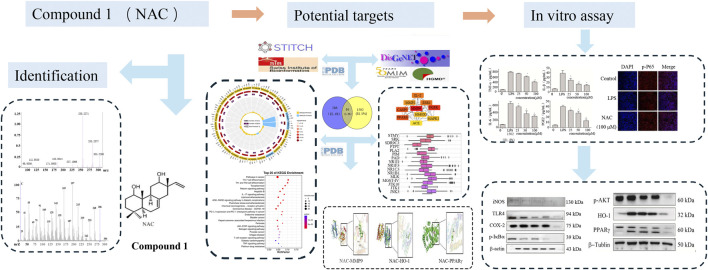
Flowchart of the study.

Compound **1** exhibited good anti-inflammatory activity; however, the molecular mechanism underlying its anti-inflammatory activity remains unclear. In this study, RAW264.7 cells were stimulated with LPS to construct an inflammatory cell model. When treated with compound **1**, its anti-inflammatory activity was confirmed. In this experimental model, the effect of compound **1** was evaluated on nitric oxide (NO) production and pro-inflammatory cytokines in inflammatory cells. Then, the changes in NF-κB phosphorylation were studied to elucidate its prospective mechanism of action. A flowchart of the study is shown in Figure 1.

## 2 Materials and methods

### 2.1 Chemicals and reagents

DMEM high-glucose medium, fetal bovine serum (FBS), and phosphate-buffered saline (PBS) culture media were purchased from Gibco (Grand Island, NY, USA). 3-(4, 5-Dimethylthiazol-2-yl)-2, 5-diphenyltetrazolium bromide (MTT) and dimethyl sulfoxide (DMSO) were purchased from Solarbio Science and Technology Co., Ltd. (Beijing, China). Mouse TNF-α, IL-6, and IL-1β enzyme-linked immunosorbent assay (ELISA) kits were purchased from eBioscience (San Diego, CA, USA). PGE2 was purchased from CUSABIO (Wuhan, China). LPS (*Escherichia coli* 0111: B4) was purchased from Sigma-Aldrich (St. Louis, MO, USA). The primary antibodies against TLR4, COX-2, iNOS, phospho-IκBα, β-actin, and HRP-conjugated goat anti-rabbit were purchased from Cell Signaling Technology (Beverly, MA, USA). The Reactive Oxygen Species Assay Kit was purchased from Beyotime Biotechnology (Beijing, China). Other chemicals and reagents used were of analytical grade, and Milli-Q (18.2 MΩ/cm) water was used to prepare all buffers and solutions.

### 2.2 Detection, extraction, and isolation

The air-dried *N. adenophyta* whole plant material (5.5 kg) was soaked and extracted thrice under room temperature (24 h each) in absolute ethanol, keeping the ratio of solid:liquid to 1:15. The extractives were combined and evaporated to dryness by rotary evaporator (Rotavapor® R-300, Buchi), yielding the ethanol residue (NAE; 480 g; 8.72%). The ethanol residue (10 g) was suspended in water and further fractionated with *n*-hexane (7.56% *w*/*w*) and ethyl acetate (26.9% w/w), and the water residue was left behind. Then, the filtrates were dried by a rotary evaporator, and the *n*-hexane soluble fraction (NAEH) and the ethyl acetate soluble fraction (NAEE) were retained. The crude (NAE) and the fractions (NAEH and NAEE) were analyzed by GC-MS (Gas Chromatograph 7890A System, Agilent, USA) for the target-oriented isolation and purification. The NAEH fraction was found to contain the target compound as a major constituent. This fraction was further subjected to normal silica gel column chromatography (200–300 mesh) eluted with *n*-hexane:ethyl acetate, increasing polarity order, to provide 24 fractions (Fr1–Fr24). The fractions were combined according to thin layer chromatography (TLC) detection on silica gel plates and analyzed by GC-MS, and the target molecule was found in Fr9–Fr11, eluted with *n*-hexane:ethyl acetate (7:3). The combined fraction Fr9–Fr11 was further chromatographed (100% DCM–100% MeOH, increasing order) over silica gel (200–300 mesh), resulting in a total of 61 fractions. The target compound was eluted from sub-fractions Frr7–Frr18 (100% DCM), and it was obtained in pure form (**1**). Compound **1** was further checked by GC-MS, and thus it was compared with the main GC-MS chromatogram and verified as the most abundant compound.

### 2.3 Target prediction and functional annotation

Undoubtedly, public databases are invaluable for generating hypotheses, but they show limitations of false positives and are influenced by curation biases. Thus, the proposed predictions are preliminary and require further experimental verification. The SwissTargetPrediction (http://www.swisstargetprediction.ch/) and STITCH (http://stitch.embl.de/) websites were used to predict the potential targets of compound **1**. In the STITCH and Swiss Target Prediction databases, the targets were limited to “*Homo sapiens*” and a confidence score ≥ 0.9. Targets with a confidence score of 0 were excluded to ensure reliability. Using “rheumatoid arthritis (RA)” as the keyword, rheumatoid arthritis-related genes were screened from the OMIM (http://www.omim.org/), DisGeNET (http://www.disgenet.org/), and HGMD (http://www.hgmd.cf.ac.uk/ac/index.php) databases.

### 2.4 Enrichment analysis

To investigate the Gene Ontology (GO) and the contributing signaling pathways of compound **1** in the treatment of inflammatory diseases, the hub targets were put into the Metascape (http://metascape.org/) tool to perform GO and Kyoto Encyclopedia of Genes and Genomes (KEGG) enrichment analysis. The adjusted *p-*value threshold was set to 0.05 to verify that the channel calculation results are statistically significant.

### 2.5 The hub target identification

Many common methods were used to identify the hub targets of compound **1** in inflammatory diseases. Gene Ontology (GO) semantic similarity provides the basis for functional comparison of gene products and, therefore, has been widely used in bioinformatics applications. Here, the package GOSemSim (Version 2.8.0) in R 3.5.2 was used to assess the functional similarity among GO terms and gene products (Yu et al., 2010). Based on this, the target gene intersections were selected. All the target genes were then integrated, and protein–protein interaction (PPI) data were subsequently obtained using the STRING database (Version 12.0, https://string-db.org/), with the required interaction score set to 0.9 (medium confidence). Finally, Cytoscape Software 3.7.2 was used to find the hub genes through the Degree method.

### 2.6 Molecular docking

The 2D structure of compound **1** was obtained from the PubChem database, and the 3D crystal structures of AKT1 (PDB code: 3O96), MAPK3 (PDB code: 6GES), AR (PDB code: 3V4A), CASP3 (PDB code: 3KGF), and MMP8 (PDB code: 1JH1) were downloaded from the Protein Data Bank (PDB) database (https://www.rcsb.org/). The protein structures were prepared by removing all nonreceptor atoms, including water, ions, and miscellaneous compounds. The irrelevant ligand contained in the crystal structure of the compound was removed by AutoDock Tools (Version 1.5.7). Next, protein docking with the active ligand component was performed by AutoDock (Version 4.2.6). All docking run options were set to default values. After docking, ligands of the lowest binding energy (highest affinity) were selected to visualize the ligand–protein interaction in PyMOL (Version 2.3).

### 2.7 Cell culture

The RAW264.7 cells were obtained from the China Academy of Chinese Medical Sciences and cultured in a high-glucose DMEM containing 10% FBS and (100 U/mL) penicillin/(100 μg/mL) streptomycin in an incubator at 37 °C and 5% CO_2_.

### 2.8 Cell viability assay

An MTT assay was used to detect the cell viability of RAW264.7 cells treated with compound **1**. The RAW264.7 cells were seeded into 96-well plates with a density of 10 × 10^5^ cells/mL. After being cultured for 24 h, compound **1** was added and cultured for 24 h with different concentrations (using micropipette) of 50 μM, 100 μM, 200 μM, 400 μM, and 800 μM, respectively, and 0.1% DMSO was set as a control. Then 10 μL of MTT (5 mg/mL) was added to each well for further culture (4 h). After that, the culture medium was removed, and 100 μL dimethyl sulfoxide (DMSO) was added to dissolve the crystal formed. The optical density (OD) values of each well were measured at 490 nm with a microplate reader (Tecan Group Ltd., Switzerland).

### 2.9 Measurement of nitric oxide (NO)

The RAW264.7 cells were inoculated into 96-well plates with the adjusted cell density of 10 × 10^5^ cells/mL. After being cultured in an incubator for 24 h, different concentrations of compound **1** (25 μM, 50 μM, and 100 μM) prepared with medium containing lipopolysaccharide (LPS) (200 ng/mL) were added, respectively. Six parallel wells were set for each concentration. Meanwhile, the blank control group and the LPS model group were set, while dexamethasone (Sigma-Aldrich, St. Louis, MO, USA) (50 µg/mL) was placed as a positive control. After the 96-well plates were again incubated for further culture for 24 h, 50 μL of the supernatant was transferred from each well into the new 96-well plates, and Griess reagent was added to each well. The absorbance of the 96-well plates was measured at 540 nm with a microplate analyzer.

### 2.10 ELISA assay

RAW264.7 cells (1 × 10^6^ cells/mL) were inoculated into 48-well plates (each well 100 μL) for 24 h in a control group with the model group (treated with 200 ng/mL LPS) and three dose groups (200 ng/mL LPS + 25 μM, 50 μM, and 100 μM of compound **1**). The cell culture supernatant (30 μL/well) was taken for the determination of cell inflammatory factors. The inhibitory effect of compound **1** on TNF-α, IL-6, and IL-1β was estimated by an ELISA kit (eBioscience, San Diego, CA, USA) according to the operation instructions.

### 2.11 Immunofluorescence assay

RAW264.7 cells were seeded in laser confocal dishes for 12 h, and then the culture was stimulated with LPS and incubated with 100 µM of compound **1**. After 24 h, the cells were washed with phosphate-buffered saline (PBS) and fixed in 4% formaldehyde for 10 min at room temperature. Afterward, the cells were permeabilized with 0.1% Triton X-100 for 4 min and washed twice with PBS. The cells were incubated with anti-NF-κB p65 antibodies (1:1,000) overnight, then washed twice with PBS and incubated with Cy3-labeled Goat Anti-Rabbit IgG for 1 h at room temperature in the dark. Finally, the cells were incubated with DAPI (4′,6-diamidino-2-phenylindole) mounting medium for 10 min. The samples were then analyzed through fluorescence microscopy (Nikon, Japan).

### 2.12 Intracellular ROS assay

The intracellular reactive oxygen species (ROS) were determined by using the Reactive Oxygen Species Assay Kit (Beyotime Biotechnology, China). In brief, RAW264.7 cells were seeded into a 6-well plate (1 × 10^5^ cells/well) for 24 h, and then they were stimulated with LPS and incubated with 100 µM (compound **1**) for 24 h. 2,7-Dichlorofluorescein diacetate (DCFH-DA) was added with a final concentration of 10 μM and incubated for another 30 min. The fluorescence intensity was examined with a fluorescence microscope (Nikon, Japan).

### 2.13 Western blot analysis

RAW264.7 cells (10 × 10^5^ cells/mL) were inoculated in 6-well plates with 2 mL per well. After 24 h of culture, the cells were treated with LPS (200 ng/mL) and different concentrations of compound **1** (25 μM, 50 μM, and 100 μM). The control group and the LPS model group were set. After 24 h, the total protein of the cells was extracted. The cells were washed with PBS, and then radio-immune precipitation assay (RIPA) lysate containing protease inhibitor and phosphatase inhibitor was added and lysed on ice for 30 min. The supernatant was obtained by centrifugation at 12,000 r/m for 10 min. The protein concentration was detected with a bicinchoninic acid (BCA) protein concentration determination kit, and the loading buffer (5x) was added and boiled for denaturation. The same concentration of protein from different groups was isolated on sodium dodecyl sulfate-polyacrylamide gel electrophoresis (SDS-PAGE) and transferred to the polyvinylidene fluoride (PVDF) membrane. The membrane was added to 4% (*w*/*v*) skim milk powder (both diluted with TBST, a mixture of Tris-buffered saline and polysorbate 20) for sealing, put in a room temperature shaker for 1 h, and then washed with TBST for 5 min. Primary antibody was added (a diluent of the primary antibody was diluted at 1:1,000) and incubated overnight in a shaker at 4 °C. The primary antibody was recycled, and the membrane was washed with TBST. Then, 4% (w/v) skim milk powder diluted secondary antibody (HRP combined with goat anti-rabbit) was added and incubated on a constant temperature shaker at 37 °C for 1 h. Finally, the samples were washed with TBST three times and detected by using electrochemiluminescence (ECL) with a ChemiScope 6000 Exp machine (Clinx, China).

### 2.14 Statistical analysis

The data were expressed as means ± standard error of the mean. Statistical analyses were performed using SPSS Version 21.0 software, utilizing an independent samples t-test and one-way ANOVA. A significance level of *p* < 0.05 was considered statistically significant.

## 3 Results

### 3.1 Isolation and structure determination

**FIGURE 2:**
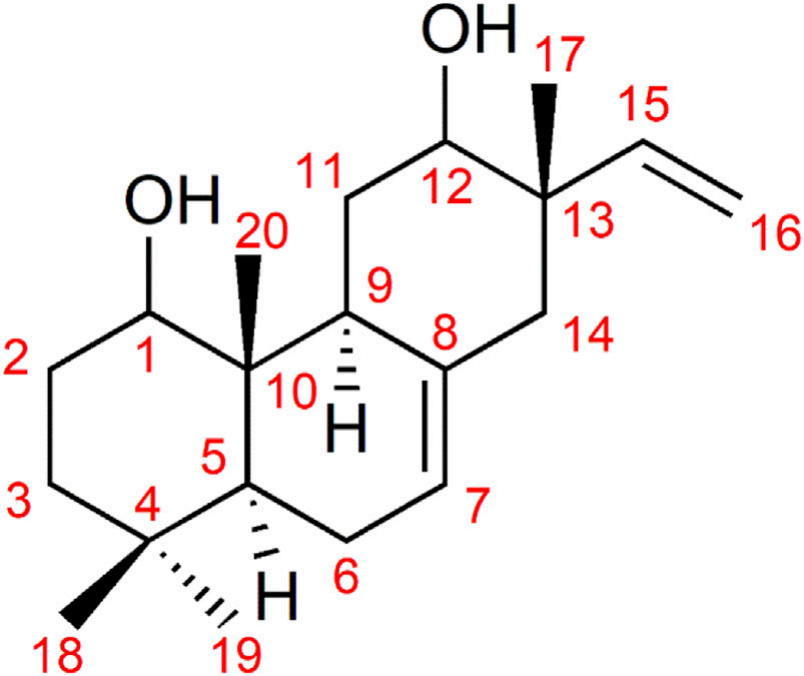
Structure of pimarane diterpenoid (**1**).

The structure of the target compound (**1**) is depicted in Figure 2. The GC spectra of the NAE (Supplementary Figure 1A), the NAEH (Supplementary Figure 1B), and the pure compound **1** (Supplementary Figure 1C) contained the target compound. These represent the extracted spectrum of the target compound, showing mass peaks at *m/z* 304 and *m/z* 286 corresponding to C_20_H_32_O_2_ and C_20_H_30_O, respectively.

The pimarane diterpenoid (**1**) was obtained as a colorless powder. It showed a positive reaction to the spray reagent (ceric sulfate) on a thin-layer chromatography (TLC) plate. The compound in its pure form was further subjected to GC-MS analysis (Supplementary Figure 2). Supplementary Figure 2A represents the GC spectrum of the pure compound **1**, while the GC-MS of the pure compound is shown in Supplementary Figure 2B. The liquid chromatography-mass spectroscopy (LC-MS) profile of the compound is shown in Supplementary Figure 3. The ESI-MS (negative ion mode) gave the quasi-molecular ion peak at *m/z* 303.2260 [M-H]^+^ (Supplementary Figure 3) corresponding to C_20_H_31_O_2_.

The ^1^H-NMR (DMSO-*d*_6_, 400 MHz) and ^13^C-NMR (DMSO-*d*_6_, 100 MHz) spectral information is provided below. ^1^H-NMR (DMSO-*d*_6_, 400 MHz) *δ*: 5.90 (1H, dd, *J* = 17.6, 10.9 Hz, H-15), 5.29 (1H, m, H-7), 4.92 (2H, m, H-16), 3.56 (1H, dd, *J* = 11.5, 4.4 Hz, H-12), 3.48 (1H, m, H-1), 2.54 (1H, m, H-9), 1.97 (2H, m, H-14), 1.85 (2H, m, H-6), 1.61 (2H, m, H-3), 1.57 (2H, m, H-11), 1.44 (2H, m, H-2), 1.36 (1H, m, H-5), 0.89 (3H, s, H-18), 0.84 (3H, s, H-19), 0.78 (3H, s, H-20), 0.75 (3H, s, H-17). ^13^C-NMR (DMSO-*d*_6_, 100 MHz) *δ*: 148.5 (CH, C-15), 135.3 (C-8), 120.5 (CH, C-7), 111.3 (CH_2_, C-16), 73.9 (CH, C-12), 69.4 (CH, C-1), 45.2 (CH_2_, C-14), 43.8 (CH, C-5), 42.0 (C-13), 41.3 (CH, C-9), 39.0 (C-10), 34.1 (CH_2_, C-3), 33.8 (CH_3_, C-19), 32.5 (C-4), 28.5 (CH_2_, C-11), 25.9 (CH_2_, C-2), 23.4 (CH_2_, C-6), 23.0 (CH_3_, C-18), 15.4 (CH_3_, C-17), 15.2 (CH_3_, C-20).

The spectral information was in agreement with reported values (Jabbarzadeh and Eslambolchimoghada, 2019; Ilyas et al., 2023), and the structure of the pimarane diterpenoid was deduced as (2*R*,4a*S*,4b*S*,8a*S*)-2-ethenyl-1,2,3,4,4a,4b,5,6,7,8,8a,9-dodecahydro-2,4b,8,8-tetramethyl-3,5-phenanthrenediol (**1**), with the molecular formula C_20_H_32_O_2_.

### 3.2 Potential target screening

**FIGURE 3:**
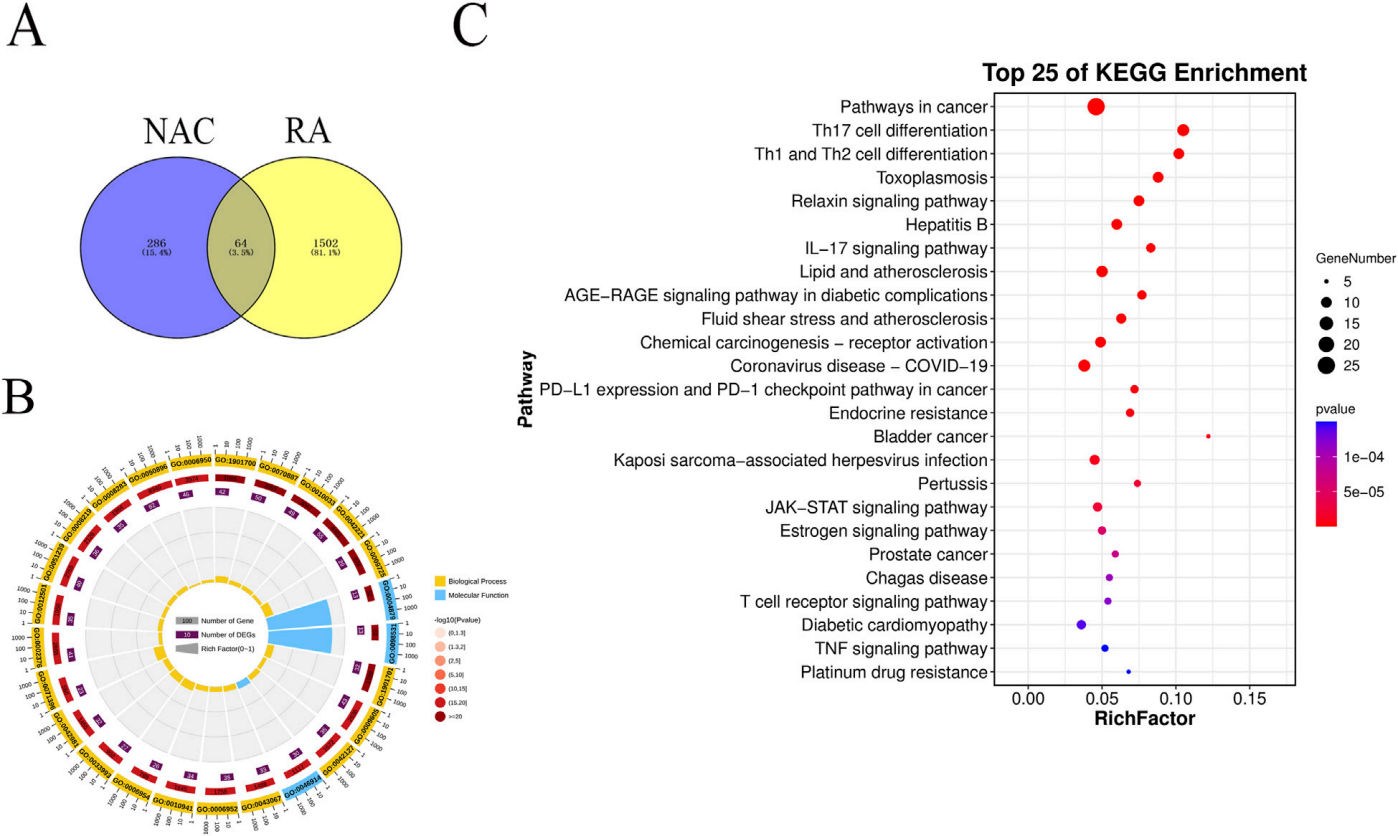
Results of overlapping genes and enrichment analysis. **(A)** Overlapping genes between NAC and RA. **(B) **GO analysis of biological process (BP) terms and molecular function. **(C)** KEGG pathway analysis.

The Swiss Target Prediction (http://www.swisstargetprediction.ch/) and STITCH (http://stitch.embl.de/) databases were used to predict targets of compound **1**. The targets were limited to *H. sapiens*, and 350 targets were collected. Then, 1,567 genes from disease databases were obtained. Finally, 64 overlapping genes were achieved (Figure 3A).

### 3.3 Biological and functional analysis

GO functional enrichment analysis and KEGG pathway enrichment of 64 potential targets were obtained through the OmicShare website (https://www.omicshare.com/tools/). As shown in Figure 3B, the enriched circle plot was used to visualize the enrichment results of the top 25 GO terms, including three (12%) molecular functions (MFs) and 22 (88%) biological processes (BPs). The outermost circle shows the classification of GO enrichment. The yellow represents BPs, and the blue represents MFs. The second circle shows the *p*-value; the smaller the value, the darker the color. The third circle shows the total number of foreground genes. The fourth circle shows the rich factor value of each classification. The BP terms, such as GO:0043167, GO:0003824, and many other terms, mainly focus on cell growth and metabolism and immune mechanisms, especially the immune response, which means these rich genes play a core regulatory role in maintaining the stability of the internal environment and responding to various stress stimuli. The top 25 ranking pathways are shown in Figure 3C. The main inflammatory pathways include IL-17 signaling, the JAK–STAT signaling pathway, the estrogen signaling pathway, and the TNF signaling pathway, along with some related to cancer. Among them, pathways in inflammation involved many genes, including IL-2, MMP1, JNK, ERK, iNOS, EGFR, and PPAR.

### 3.4 Hub genes

**FIGURE 4:**
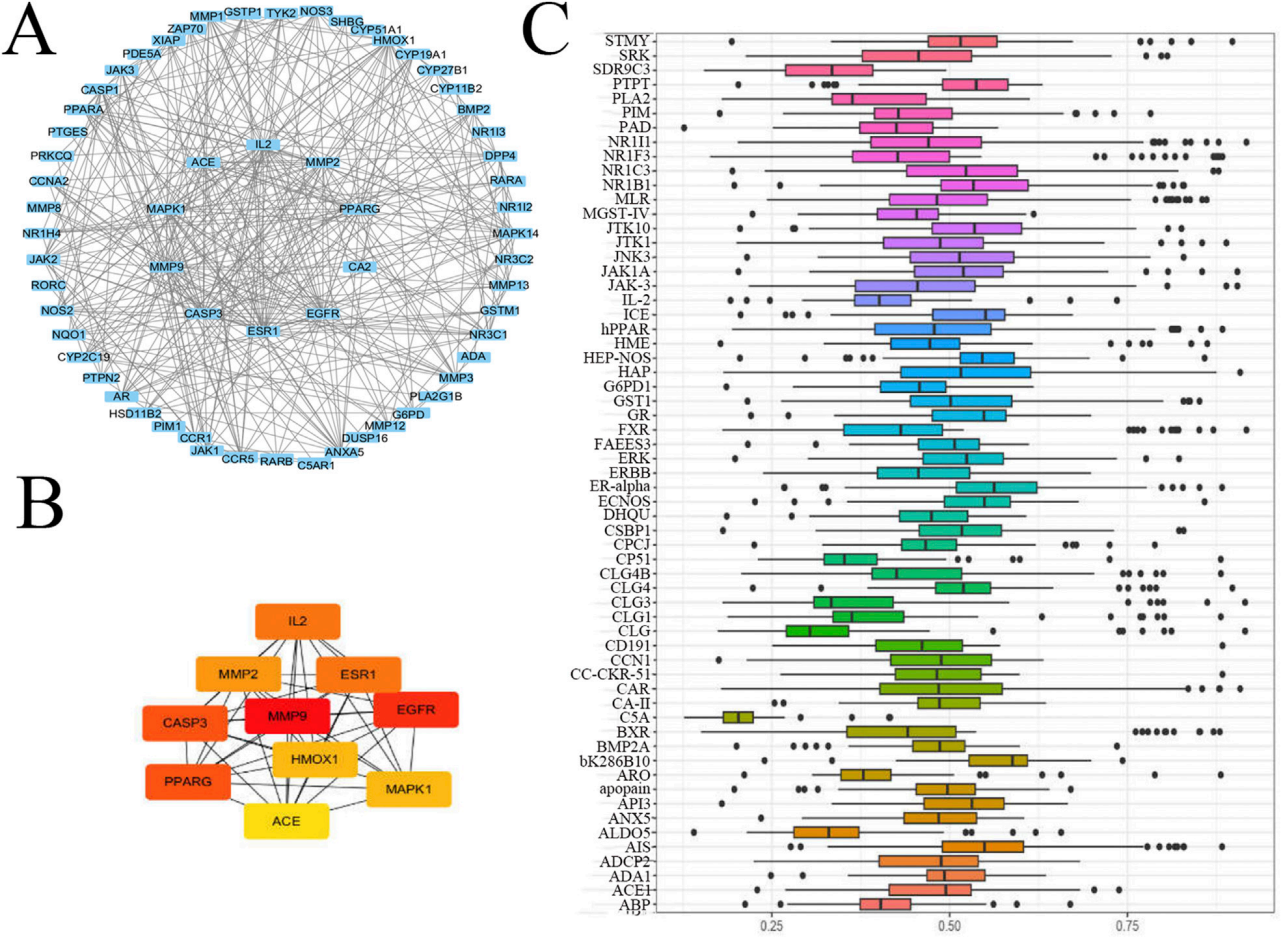
Hub gene analysis. **(A)** PPI network. The insect genes were input into Cytoscape, and a network was displayed. **(B)** The core genes were obtained by using the Degree function in Cytoscape software 3.7.2. **(C)** The core genes were obtained using the package GOSemSim (Version 2.8.0) in R 3.5.2.

In order to obtain the hub genes, the PPI network was established to better understand the connection between these proteins (Figure 4A). Disconnected nodes are not shown in this figure, and the confidence score is set to 0.90. The final network embodied 64 nodes and 156 edges. Then, the Degree method in Cytoscape software 3.9 was used to evaluate the importance of proteins (Figure 4B). From the result, *IL-2*, *ESR1*, *MAPK1*, *EGFR*, *ACE*, *PPARγ*, *HO-*1, *MMP2/9*, and *CASP3* have shown the core relation. The package GOSemSim was used to assess gene similarity and obtain the hub genes (Figure 4C), which are *ERα*, *HO-1*, *iNOS*, *PPARγ*, *MMP3/9*, *CLG4*, and *GR*. From the above results, all these 15 targets are related to the treatment of inflammatory diseases, especially some overlapping proteins, such as *MMP9*, *HO-1*, and *PPARγ*.

### 3.5 Docking

**FIGURE 5:**
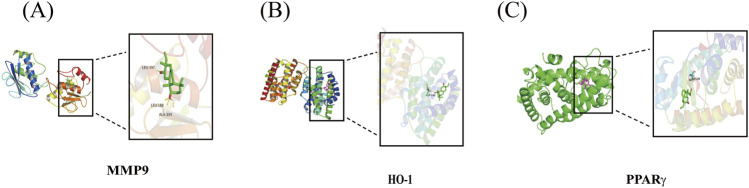
Partial diagram of molecular docking: **(A)** NAC-MMP9, **(B)** NAC-HO-1, and **(C)** NAC-PPARγ.

Through the above results, the overlapping three proteins were selected for molecular docking with compound **1** using AutoDock software. The employed molecular docking uses computational forecasting to evaluate the potential interaction between compound **1** and the selected receptors. The details of the docking results are shown in Figure 5 and Table 1. From the results, these three targets were predicted as potential receptors for **1** to treat inflammatory diseases. Our network analysis predicted, and molecular docking reinforced, the potential of the hydroxyl group in compound **1** to interact and form conventional hydrogen bonds with LEU-397, LEU-188, and ALA-189 on MMP9, to bond with ASP-140 on HO-1, and to bond with SER-289 on PPARγ.

**TABLE 1:** Binding energy.

No.	Protein	PDB id	Energy/kcal·mol^−1^
1	MMP9	1GKC	−8.05
2	HO-1	1N3U	−7.24
3	PPARγ	9CK0	−7.76

### 3.6 The cell viability of compound **1**

**FIGURE 6:**
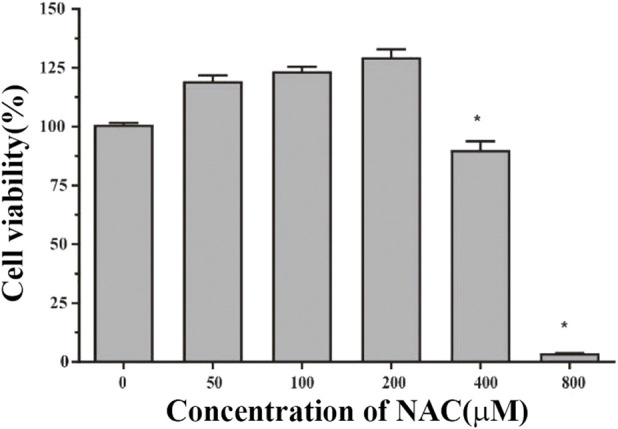
Effects of compound **1** on the viability of RAW264.7 cells. The cell viability of NAC in different concentrations (0, 25 μM, 50 μM, 100 μM, and 200 μM) for 24 h was determined by the MTT method. The results are presented as the mean ± SEM; *p < 0.05 versus untreated control.

To evaluate the toxicity level of compound **1**, an MTT assay was first conducted using the RAW264.7 cells. As shown in Figure 6, the results showed that compound **1** did not induce cell death below 200 µM concentration. Above 200 μM, it has a significant toxic effect on the cell (*p* < 0.05).

### 3.7 Effect of compound **1** on NO production

**FIGURE 7:**
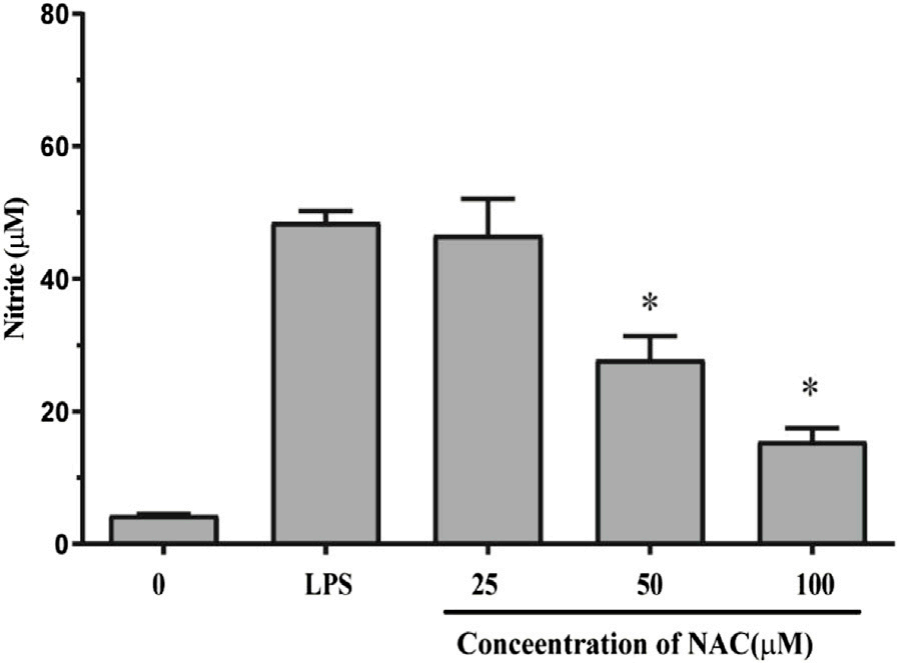
NO inhibitory effects of **1** on RAW264.7 cells. The NO inhibitory effect of NAC in different concentrations (0 µM, 25 µM, 50 µM, and 100 μM) for 24 h was determined by the Griess method. The results are presented as the mean ± SEM; *p < 0.05 versus untreated control.

To evaluate the NO production of compound **1**, a Griess assay was conducted using the RAW264.7 cell. As shown in Figure 7, the results showed that the NO production decreased in a dose-dependent fashion from 0–100 μM.

### 3.8 Inhibitory effect of pro-inflammatory cytokines

**FIGURE 8:**
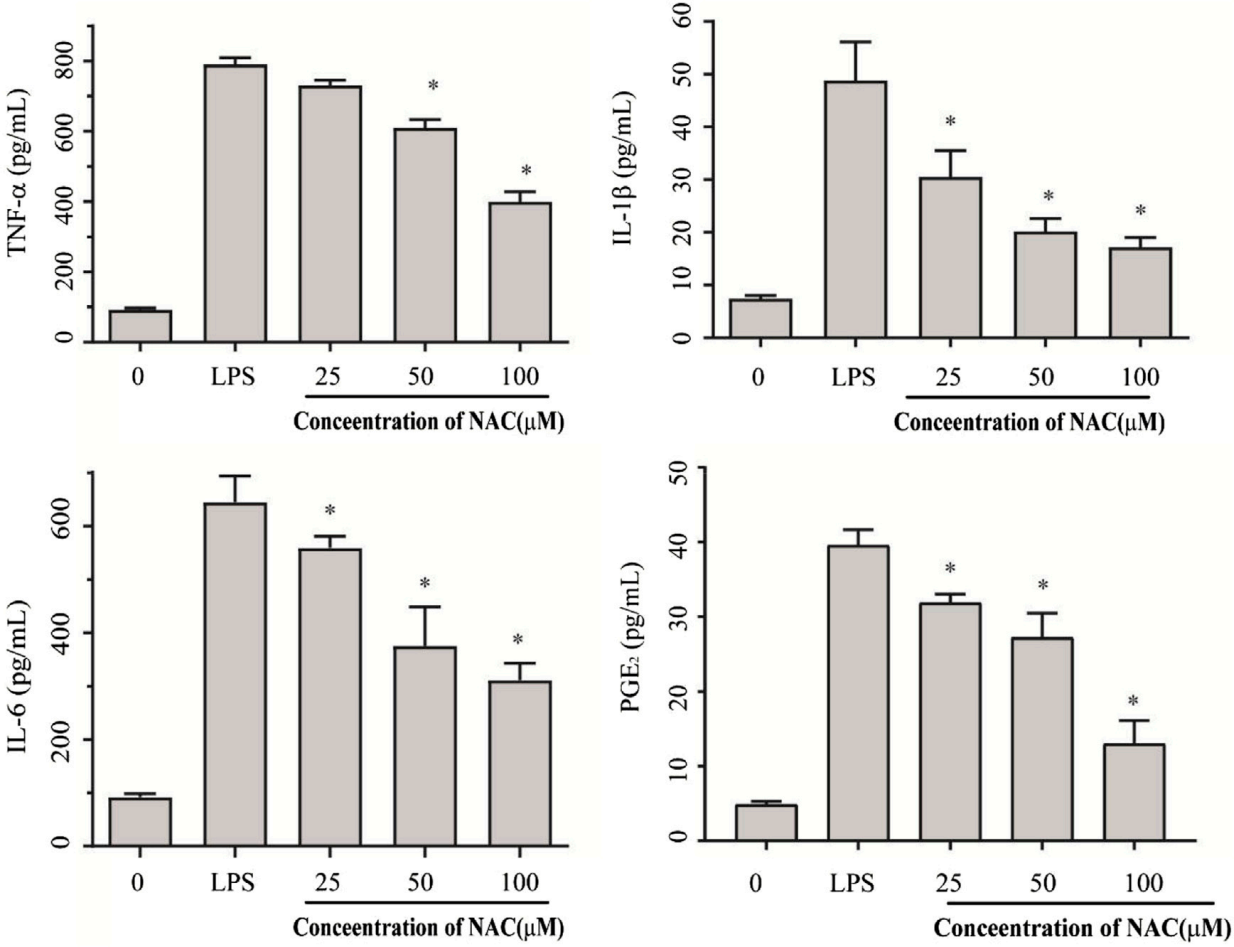
The effect of compound **1** on IL-1β, IL-6, TNF-α, and PGE2 production in LPS-treated RAW264.7 cells. The concentrations of IL-1β, IL-6, TNF-α, and PGE2 production in the culture medium were determined by ELISA. All data were obtained from three independent biological replicates of the experiment. The results are presented as the mean ± SEM; *p < 0.05 versus model group.

To investigate the effect of compound **1** on LPS-induced inflammatory responses, LPS with three concentrations of compound **1** (25 µM, 50 µM, and 100 µM) was added to a 48-well cell plate. Production of IL-6, IL-1β, TNF-α, and PGE2 was noted. The results shown in Figure 8, compared with the control group, PGE2, TNF-α, IL-1β, and IL-6, are significantly decreased with the increase in doses (*p* < 0.05). It also has a dose-dependent effect. Compound **1** significantly suppressed pro-inflammatory mediators, especially IL-1β, which showed a sharp loss in activity at 25 µM.

### 3.9 Effects of compound **1** on NF-κB p65 in LPS-stimulated RAW264.7 cells

**FIGURE 9:**
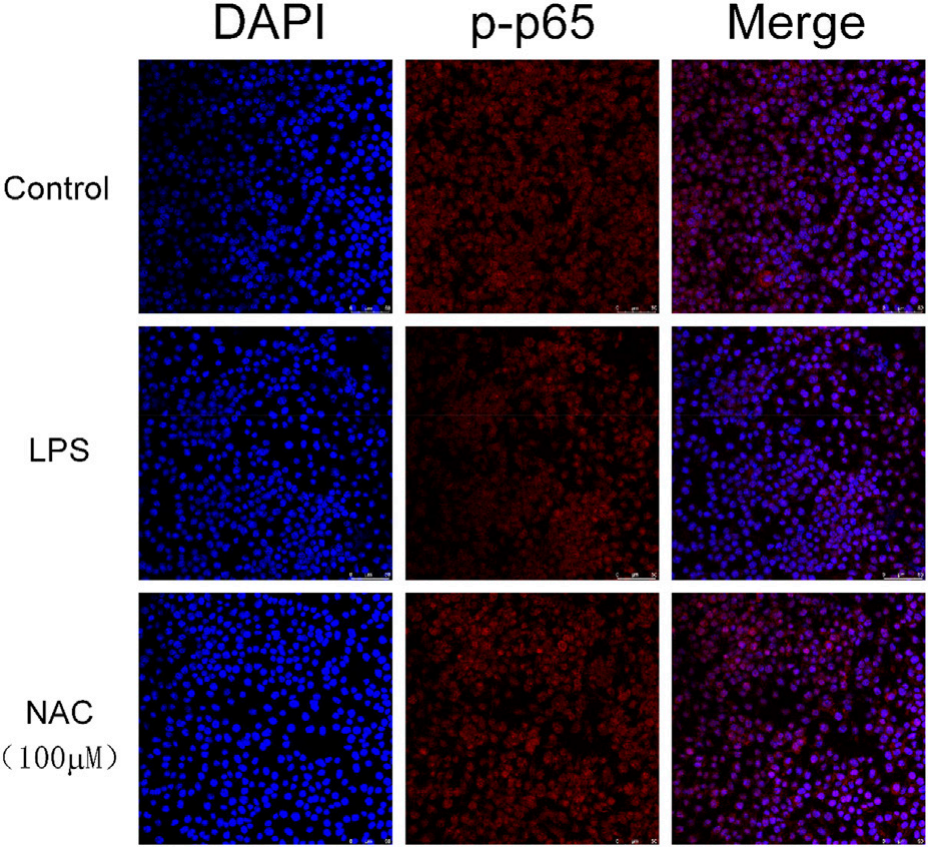
The location of the p65 subunit of NF-κB was tested through the immunofluorescence method. According to the kit steps, cells were seeded into 20 mm confocal small dishes for 24 h, then the control, LPS, and LPS + NAC (100 μM) groups were incubated for 24 h. All the groups were fixed, incubated with anti-NF-κB p65 antibodies overnight at 4 °C, and incubated with Cy3-labeled Goat Anti-Rabbit IgG for 1 h at room temperature. Finally, DAPI mounting medium was added. The images were captured using a microscope.

The NF-κB signaling pathway is an important inflammatory response pathway. As shown in Figure 9, LPS treatment resulted in the translocation of NF-κB from the cytoplasm to the nucleus, as indicated by the detection of the Cy3-conjugated anti-NF-κB antibody and the blue fluorescence of nuclear counterstain DAPI. In contrast, the translocation of NF-κB was reduced in cells treated with compound **1**, which means it can effectively induce the translocation of NF-κB p65.

### 3.10 Intracellular ROS assay

**FIGURE 10:**
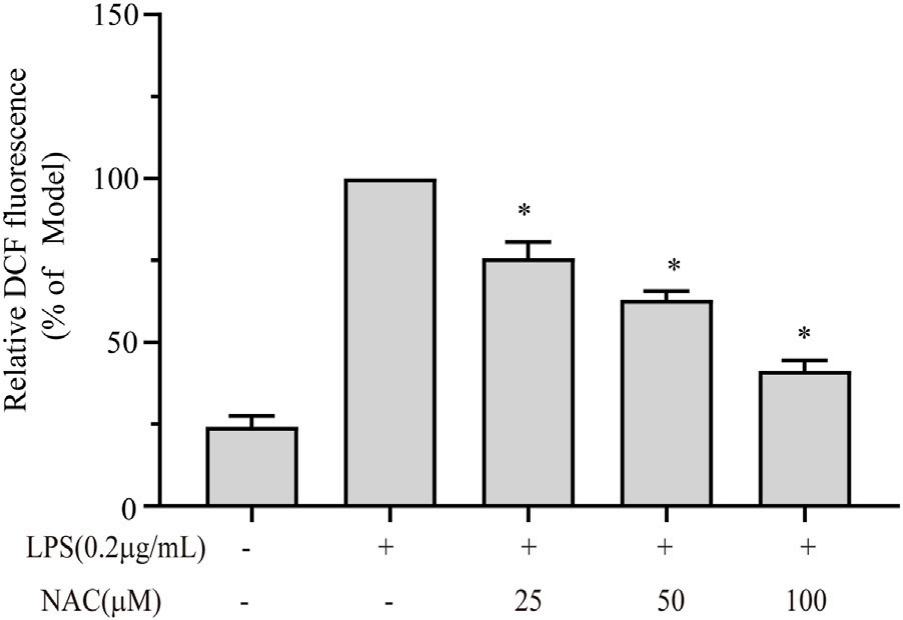
NAC can inhibit LPS-induced ROS production. The fluorescence intensity was detected using a fluorescence microscope. The results are presented as the mean ± SEM; *p < 0.05 versus model group.

Excessive ROS can increase inflammation through the activation of a series of protein kinases, inflammatory transcription factors, and phosphorylated protein kinases. In turn, it can also produce more ROS. To investigate whether **1** can alleviate the amount of LPS-induced ROS production, the molecular probe DCFH-DA was applied. As shown in Figure 10, the intracellular ROS level was induced by LPS treatment, and **1** significantly inhibited the ROS production, especially when the concentration reached 100 μM.

### 3.11 Inhibitory effect of compound **1** against LPS-stimulated RAW264.7 cells by inhibiting the NF-κB and PPARγ signal pathways

**FIGURE 11:**
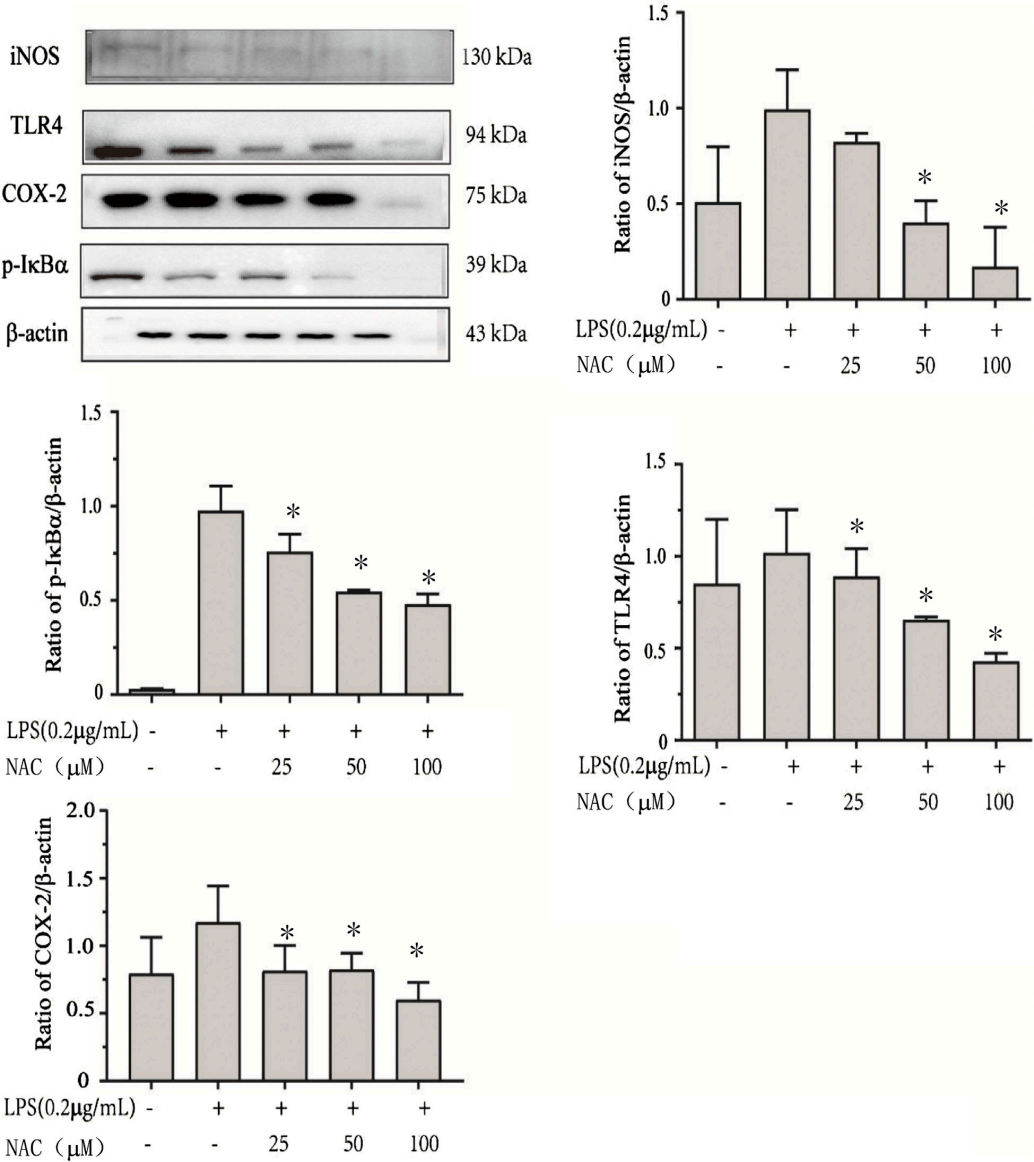
The effects of compound **1** on the protein expression connected with the inhibition of the NF-κB signaling pathway. After RAW264.7 cells were seeded into 6-well plates for 24 h, different concentrations (25–100 μM) of **1** with LPS were added for another 24 h. Then, the protein was extracted by RIPA, detected by a BCA kit, and the expression of proteins associated with the inhibition of NF-κB was detected by a Western blot analysis. Comparison of the levels of protein relative to the level of β-actin. Data are presented as the mean ± SEM of triplicate tests. *p < 0.05 versus model group.

In normal cells, NF-κB, combined with IκBα as a protein complex, is located in the cytoplasm. After stimulation by LPS, its inhibitor IκBα was phosphorylated, and then it was ubiquitinated, resulting in proteolytic degradation and releasing NF-κB to translocate to the nuclei and inducing an inflammatory reaction. Therefore, Western blot analysis was performed to explore the effect of compound **1** on the phosphorylation and degradation of IκBα through the NF-κB signaling pathway. From the results of this assay, it was found that **1** strongly inhibited the NF-κB-dependent inflammation pathway. As shown in Figure 11, the IκBα phosphorylation level was significantly increased in the LPS-treated group, but this effect was reduced significantly with the dose of **1** (*p* < 0.05).

**FIGURE 12:**
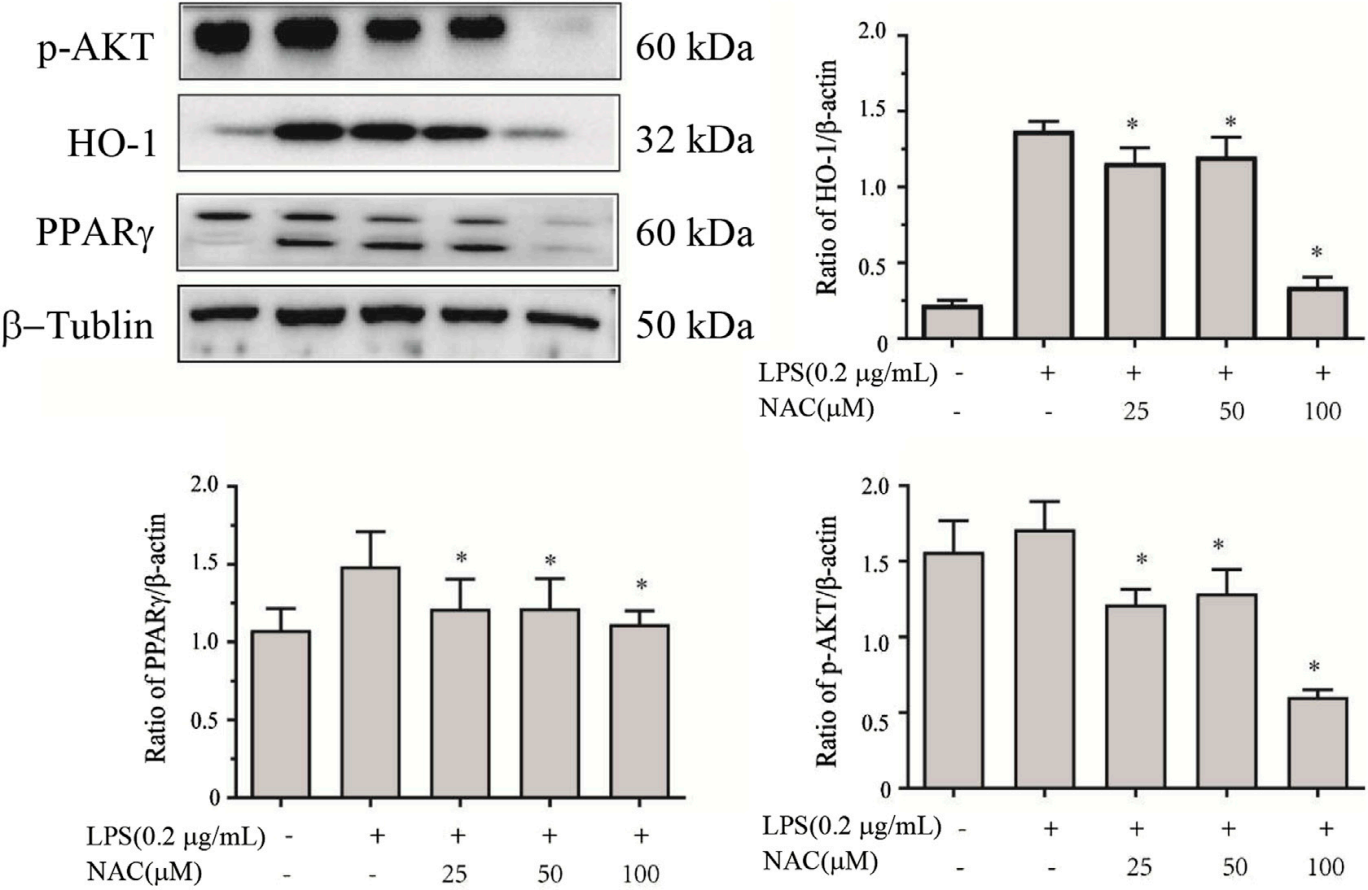
The effects of compound **1** on the protein expression connecting with the inhibition of the AKT signaling pathway. RAW264.7 cells were used, as described in Figure 10. The total and phosphorylated protein kinases were detected by Western blot analysis using their corresponding antibodies. Data are presented as the mean ± SEM of triplicates. *p < 0.05 versus model group.

**FIGURE 13:**
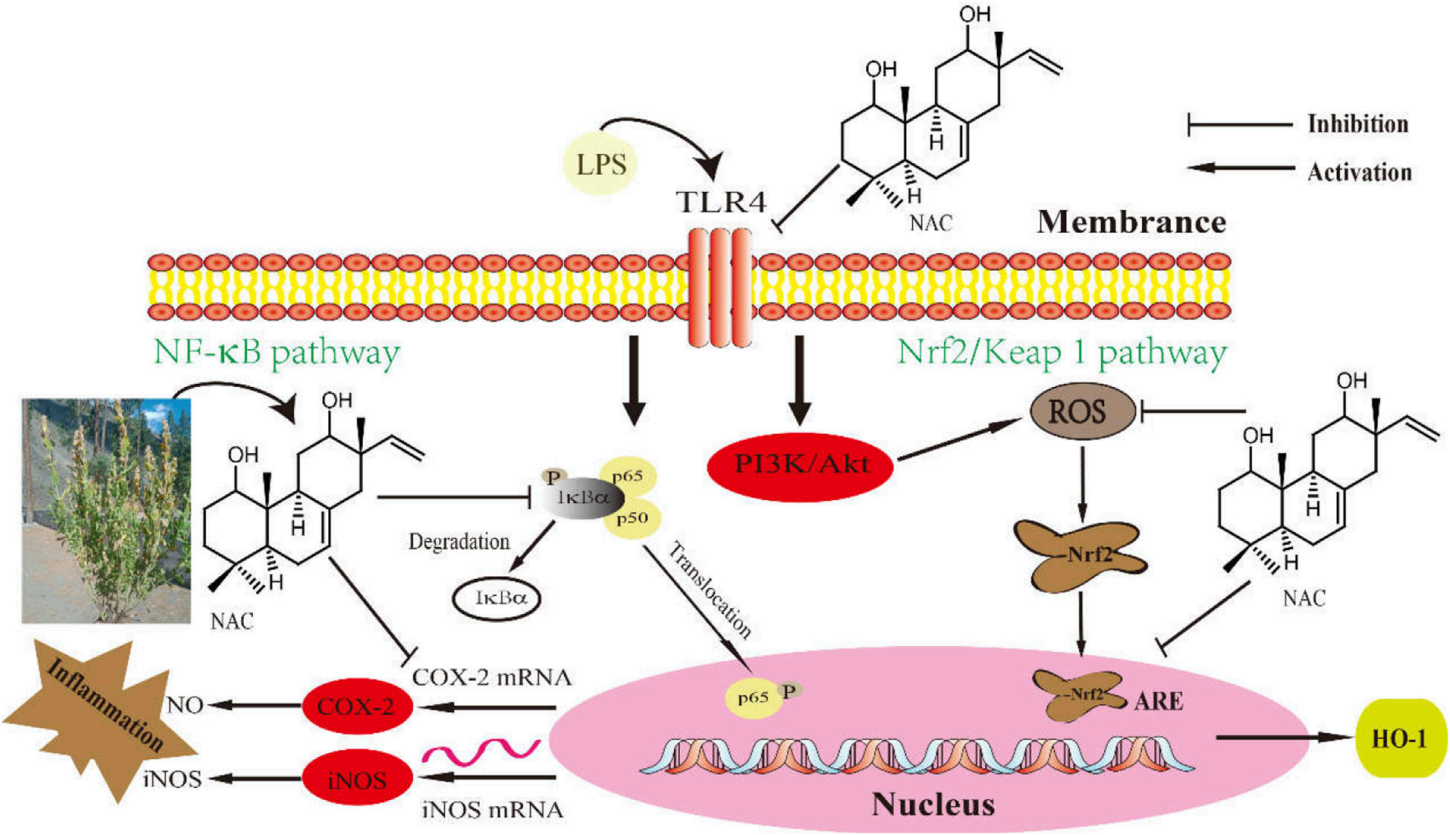
The molecular mechanism underlying the anti-inflammatory effect of **1** (NAC) on LPS-induced RAW264.7 cells. NAC can effectively inhibit the LPS-induced COX-2 and iNOS expression, which is largely dependent on the suppression of the IκB-α/NF-κB pathways. In addition, it is responsible for both the production of excessive ROS and for the overexpression of PPARγ.

Many studies have shown that many other signaling pathways are closely related to NF-κB. PPARγ is a major molecule for NF-κB activation and for inflammatory gene expression. We examined these upstream signaling pathways by carrying out Western blot analysis to detect the protein levels of AKT, HO-1, and PPARγ. When cells were treated with LPS alone, they increased markedly. Treatment with **1** in LPS-induced cells showed that compound **1** can block the PPARγ pathway, which regulates downstream iNOS and COX-2 gene expression, as shown in Figure 12. The possible mechanisms of both NF-κB and PPARγ pathways are described in Figure 13.

## 4 Discussion

Many *Nepeta* species have medicinal value and are used extensively, particularly in India, Pakistan, and Iran. The plants are used to treat a variety of ailments and conditions like chicken pox, tuberculosis, malaria, pneumonia, influenza, measles, stomach disorders, eye complaints, respiratory disorders, asthma, colds, and coughs. *Nepeta* species possess a variety of pharmacological activities, namely, anti-inflammatory, anti-nociceptive, anti-Alzheimer, anticancer and cytotoxic, antioxidant, immunomodulatory, antimicrobial, antifungal, insecticidal, and other biological activities. This may be attributed to its various components, such as terpenoids, volatiles, and many others (Sharma et al., 2021). Many pimarane diterpenoids have been reported for pharmacological activities, such as antioxidant, antitumor, anti-inflammatory, and antibacterial (Reveglia et al., 2018).

Network analysis has developed rapidly and has become a research strategy for new drug discovery and creation. By combining the advantages of assay research, it can fully illustrate the unclear pharmacological mechanisms and unknown targets. In this research, we utilized network analysis to generate a hypothesis that compound **1** might treat inflammatory diseases through multiple targets, such as MMP9, HO-1, and PPAR. This hypothesis was then experimentally tested in the following assays. Inflammatory cell exposure to endotoxins and cytokines produces excessive NO by iNOS, which has been linked to the progression of inflammatory diseases and carcinogenesis (Ohshima and Bartsch, 1994). Another important enzyme, COX-2, and its product prostaglandin also promote cell proliferation, tumor angiogenesis, and growth. Recently, compound **1** has been reported for wound healing and angiogenesis (Jabbarzadeh and Eslambolchimoghada, 2019) and also for cancer treatment (Jabbarzadeh and Taylor, 2020).

In this study, for the first time, we focused on the inflammatory effect of compound **1,** which was isolated (GC-MS guided) for the first time from *N. adenophyta* Hedge, an herbal remedy for ethnogynecological issues, and demonstrated that it could inhibit NO, TNF-α, IL-1β, PGE2, and IL-6 significantly in RAW264.7 cells. Many stimuli, such as cytokines and lipopolysaccharide (LPS), can induce excessive generation of NO, TNF-α, and IL-6. All the pro-inflammatory cytokines, which can promote cell proliferation, tumor angiogenesis, and growth, have been linked to the onset of inflammatory diseases and some other diseases (Ohshima and Bartsch, 1994; Meric et al., 2006). The present data show that compound **1** strongly and dose-dependently inhibits LPS-induced NO and PGE2 production by decreasing the protein expression of iNOS and COX-2, which agrees with previous observations.

Several protein kinases have been reported to regulate nuclear transcription factor NF-κB and PPARγ pathways. According to the previous reference studies, under the inflammatory state of macrophages induced by lipopolysaccharide, the two molecules form a dynamic antagonistic relationship during the inflammatory process. The activation of NF-κB usually promotes the release of pro-inflammatory factors (such as TNF-α, IL-1β, and IL-6), while PPARγ, as a nuclear receptor, can exert anti-inflammatory effects by inhibiting the nuclear translocation of NF-κB or by binding to its co-inhibitory factors (Corripio-Miyar et al., 2007). Although PPARγ is an anti-inflammatory transcription factor, its stable expression and activity may require specific signals, such as the activity of p-AKT. Our compound **1** has the potential to inhibit AKT in a previous network. It also shows suppression of an upstream signal, p-AKT, as observed in our Western blot results, which is necessary for maintaining the expression of PPARγ. So, as we can see, the decreased stability caused by p-AKT inhibition induces decreased expression of PPARγ.

Our results show that compound **1** blocked LPS-induced phosphorylation of typical PPARγ, and further study of the above relative protein indicated that the protein levels of HO-1 and AKT can be inhibited. The inhibition of these specific mediators observed *in vitro* holds significant implications for potential *in vivo* anti-inflammatory effects, particularly concerning systemic inflammation. Macrophages are pivotal sentinel cells within the innate immune system and major contributors to the cytokine cascade that characterizes systemic inflammatory responses (Stephen et al., 2008). TNF-α, IL-6, and IL-1β are not merely markers of localized macrophage activation; they are central drivers of systemic inflammation. Elevated circulating levels of these cytokines are hallmarks of conditions in rheumatoid arthritis, inflammatory bowel disease, and cytokine release syndromes. The observed downregulation of cytokines suggests that compound **1** could potentially mitigate the most potent initiators of systemic inflammation *in vivo*.

Consequently, the collective inhibition of TNF-α, IL-6, IL-1β, NO, and PGE2 observed in our RAW264.7 model plausibly suggests that compound **1** possesses a multi-targeted mechanism capable of dampening the core inflammatory cascade. The *in vitro* efficacy against these specific targets provides a strong mechanistic rationale for hypothesizing beneficial effects in *in vivo* models of systemic inflammatory diseases. The observed effects could potentially translate to mitigating the hyper-inflammatory state, reducing end-organ damage, and improving survival outcomes in scenarios like septic shock or alleviating chronic systemic inflammation in autoimmune disorders.

However, it is crucial to emphasize that the effects *in vivo* remain speculative, and the RAW264.7 model has inherent limitations. It cannot recapitulate the complex pharmacokinetics, biodistribution, metabolism, or potential toxicity of the compound within a whole organism. Systemic inflammation involves intricate crosstalk between numerous cell types and organs. Factors such as bioavailability and effects on adaptive immunity remain unknown. Therefore, while these findings are promising and mechanistically suggestive of potential systemic benefits, definitive conclusions regarding *in vivo* efficacy and safety require rigorous validation in appropriate animal models of systemic inflammation.

## 5 Conclusion

In summary, the current study revealed that IκBα and PPARγ play a key role in LPS-induced inflammatory expression, which provides important targets and alternatives in the treatment of inflammatory diseases. Compound **1**, a natural product component from a plant widely used medicinally in Pakistan, represents a candidate for further investigation in the development of anti-inflammatory agents.

## Data Availability

The original contributions presented in the study are included in the article/Supplementary Material, further inquiries can be directed to the corresponding authors.
