# Supplementary material for: Anti-inflammatory effect of a pimarane diterpenoid isolated from Nepeta adenophyta Hedge based on a network analysis approach and experimental assessment
Source: Front Pharmacol. 2025 Dec 18;16:1652902. doi: 10.3389/fphar.2025.1652902 (PMC12756164; doi:10.3389/fphar.2025.1652902)
Supplement: Supplementary file 1 [file Supplementaryfile1.docx]

Supplementary Material


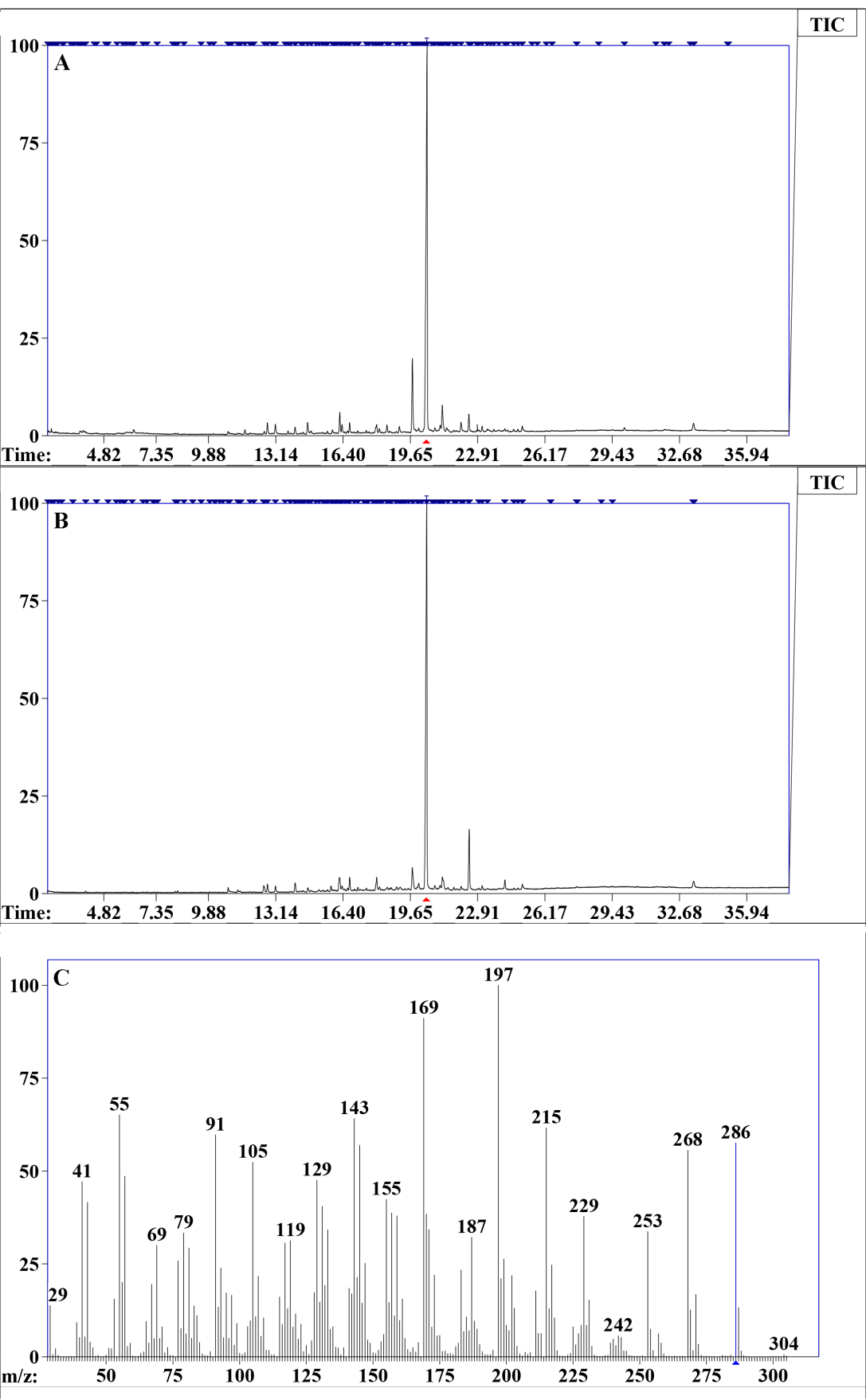


**Supplementary Figure 1.** **(A)** GC-MS/MS chromatogram of NAE. **(B)** GC-MS/MS chromatogram of NAEH. **(C)** Extracted spectrum of NAC.


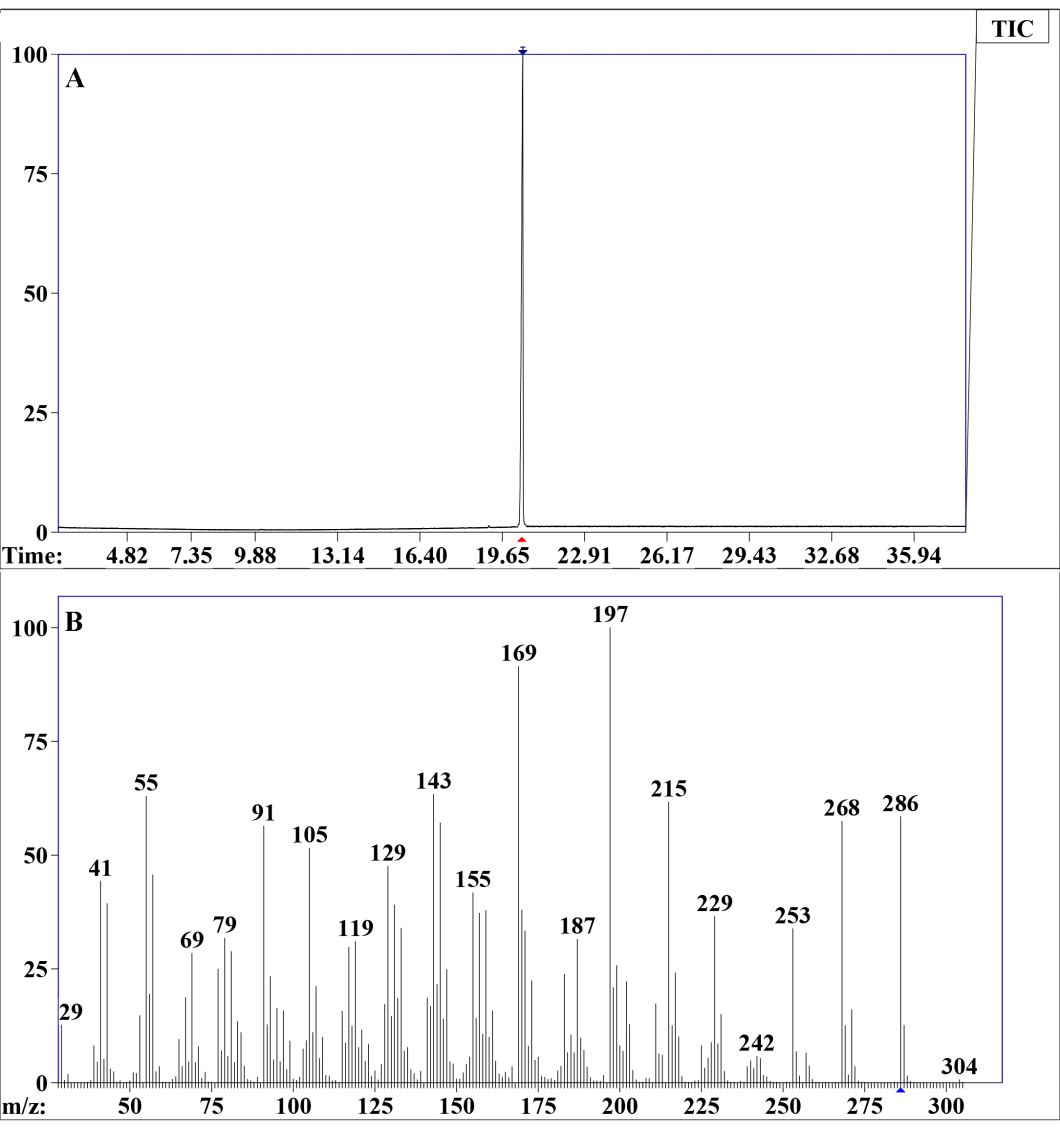


**Supplementary Figure 2.** **(A)** GC-MS/MS chromatogram of NAC. **(B)** GC-MS/MS fragmentation of NAC.


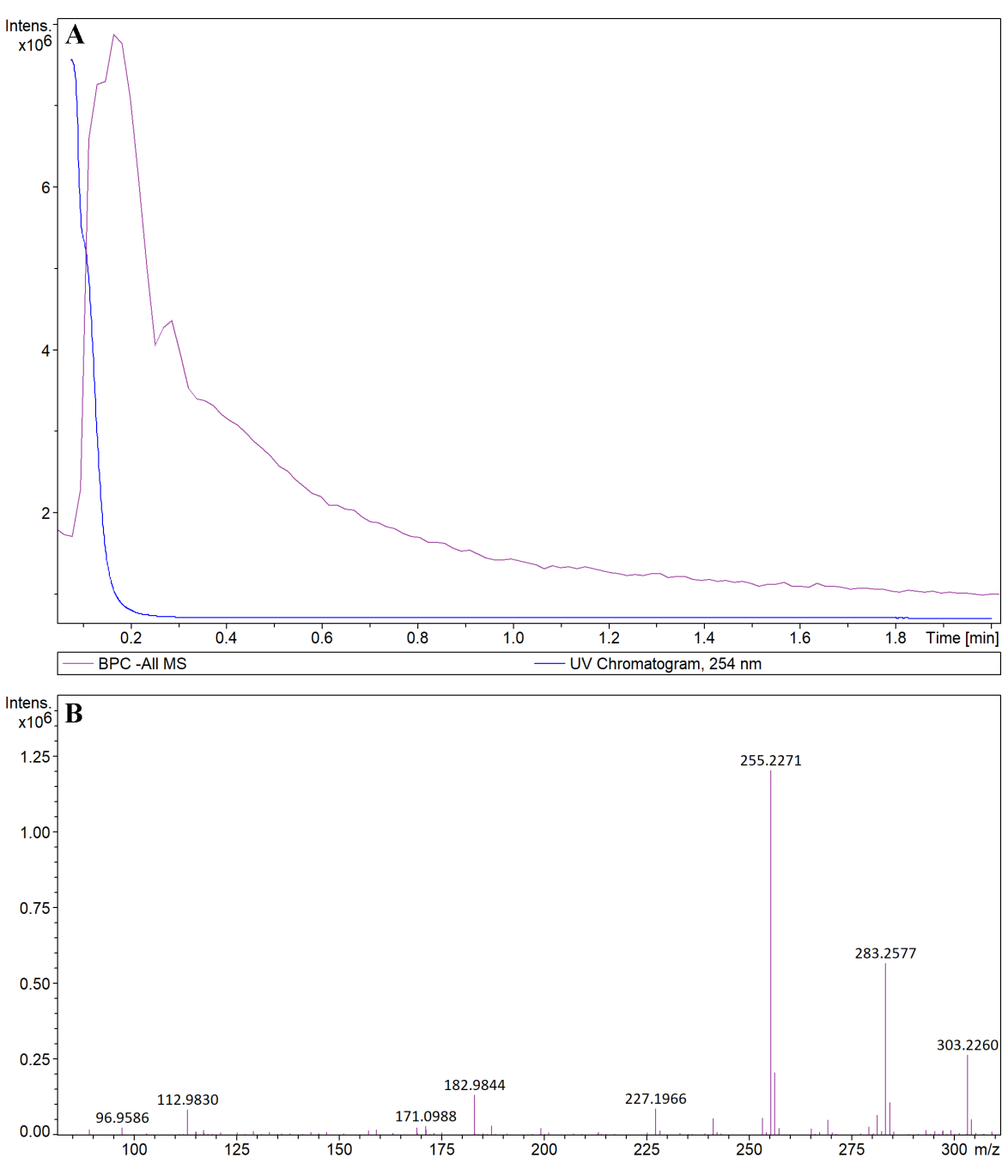


**Supplementary Figure 3.** **(A)** LC-MS/MS chromatogram of NAC. **(B)** LC-MS/MS fragmentation of NAC.
